# Supplementary material for: Cardiovascular diseases among people living with HIV/AIDS in Ethiopia: A scoping review
Source: PLoS One. 2026 May 5;21(5):e0348283. doi: 10.1371/journal.pone.0348283 (PMC13143083; doi:10.1371/journal.pone.0348283)
Supplement: S1 File — (DOCX) [file pone.0348283.s001.docx]

**Supplementary file 1: Search strategy**

**PubMed**

| Search | Key word (concepts) | Search string | Items found (13/11/2023) | Items found (13/03/2026) |
| --- | --- | --- | --- | --- |
| #1 | Cardiovascular disease | (("Cardiovascular Diseases"[Mesh]) OR ("Cardiovascular disease*"[tiab] OR "Cardio-vascular disease*"[tiab] OR "Cardio vascular disease*"[tiab] OR "Cardiovascular illness*"[tiab] OR "Cardio-vascular illness*"[tiab] OR "Cardio vascular illness*"[tiab] OR "Cardiovascular mortalit*"[tiab] OR "Cardio-vascular mortalit*"[tiab] OR "Cardio vascular mortalit*"[tiab] OR "Cardiac disease*"[tiab] OR "Coronary disease*"[tiab] OR "Cardiac arrest*"[tiab] OR "Heart disease*"[tiab] OR "Myocardial infarction"[tiab] OR Stroke[tiab] OR "Artery disease*"[tiab] OR "Vascular disease*"[tiab] OR Hypertensi*[tiab] OR Aneurysm[tiab] OR Arrhythmia[tiab] OR Atherosclerosis[tiab] OR Heart failure[tiab] OR CVD[tiab] OR "high blood pressure"[tiab] OR CHF[tiab])) | 3,308,731 | 326,380 |
| #2 | HIV/AIDS | (("Acquired Immunodeficiency Syndrome"[Mesh] OR "HIV"[Mesh]) OR (AIDS[tiab] OR "Acquired Immunodeficiency Syndrome*"[tiab] OR "Acquired Immune Deficiency Syndrome*"[tiab] OR "Acquired Immuno-Deficiency Syndrome*"[tiab] OR "Acquired Immuno Deficiency Syndrome*"[tiab] OR HIV[tiab] OR "Human Immunodeficiency Virus*"[tiab] OR "Human Immuno Deficiency Virus*"[tiab] OR "Human Immuno-Deficiency Virus*"[tiab] OR "AIDS Virus*"[tiab] OR "Acquired Immune Deficiency Syndrome Virus*"[tiab] OR "Acquired Immunodeficiency Syndrome Virus*"[tiab] OR "HIV/AIDS"[tiab])) | 482,380 | 43,798 |
| #3 | Ethiopia | ((Ethiopia[Mesh]) OR Afar[tiab] OR Amhara[tiab] OR Benishangul*[tiab] OR Gumuz*[tiab] OR Gambel*[tiab] OR Harar*[tiab] OR Oromia*[tiab] OR Somali*[tiab] OR SNNP*[tiab] OR "Southern nation nationalit*"[tiab] OR Tigra* [tiab] OR "Addis Ababa"[tiab] OR "Dire Dawa"[tiab] OR Ethiop*[tiab]) | 31,569 | 10,632 |
| **#4** | **Final** | **#1 AND #2 AND #3** | **93** | **42** |

**EMBASE**

| Search | Key word (concepts) | Search string | Items found (13/11/2023) | Items found (13/03/2026) |
| --- | --- | --- | --- | --- |
| #1 | Cardiovascular disease | (('cardiovascular disease'/exp) OR ('Cardiovascular disease*' OR 'Cardio-vascular disease*' OR 'Cardio vascular disease*' OR 'Cardiovascular illness*' OR 'Cardio-vascular illness*' OR 'Cardio vascular illness*' OR 'Cardiovascular mortalit*' OR 'Cardio-vascular mortalit*' OR 'Cardio vascular mortalit*' OR 'Cardiac disease*' OR 'Coronary disease*' OR 'Cardiac arrest*' OR 'Heart disease*' OR 'Myocardial infarction' OR Stroke OR 'Artery disease*' OR 'Vascular disease*' OR Hypertensi* OR Aneurysm OR Arrhythmia OR Atherosclerosis OR 'Heart failure' OR CVD OR 'high blood pressure' OR CHF):ab,ti) | 5,800,566 | 957,507 |
| #2 | HIV/AIDS | (('Human immunodeficiency virus'/exp OR 'Human immunodeficiency virus infection'/exp OR 'acquired immune deficiency syndrome'/exp) OR (AIDS OR 'Acquired Immunodeficiency Syndrome*' OR 'Acquired Immune Deficiency Syndrome*' OR 'Acquired Immuno-Deficiency Syndrome*' OR 'Acquired Immuno Deficiency Syndrome*' OR HIV OR 'Human Immunodeficiency Virus*' OR 'Human Immuno Deficiency Virus*' OR 'Human Immuno-Deficiency Virus*' OR 'AIDS Virus*' OR 'Acquired Immune Deficiency Syndrome Virus*' OR 'Acquired Immunodeficiency Syndrome Virus*' OR 'HIV/AIDS'):ab,ti) | 1,051,387 | 131,890 |
| #3 | Ethiopia | (('Ethiopia'/exp OR 'Ethiopian'/exp) OR (Ethiop* OR Afar OR Amhara OR Benishangul* OR Gumuz* OR Gambel* OR Harar* OR Oromia* OR Somali* OR SNNP* OR 'Southern nation nationalit*' OR Tigra* Or 'Addis Ababa' OR 'Dire Dawa'):ab,ti) | 41,279 | 13,358 |
| **#4** | **Final** | **#1 AND #2 AND #3** | **429** | **216** |

**Web of Science**

| Search | Key word (concepts) | Search string | Items found (13/11/2023) | Items found (13/03/2026) |
| --- | --- | --- | --- | --- |
| #1 | Cardiovascular disease | TS=('Cardiovascular disease*' OR 'Cardio-vascular disease*' OR 'Cardio vascular disease*' OR 'Cardiovascular illness*' OR 'Cardio-vascular illness*' OR 'Cardio vascular illness*' OR 'Cardiovascular mortalit*' OR 'Cardio-vascular mortalit*' OR 'Cardio vascular mortalit*' OR 'Cardiac disease*' OR 'Coronary disease*' OR 'Cardiac arrest*' OR 'Heart disease*' OR 'Myocardial infarction' OR Stroke OR 'Artery disease*' OR 'Vascular disease*' OR Hypertensi* OR Aneurysm OR Arrhythmia OR Atherosclerosis OR 'Heart failure' OR CVD OR 'high blood pressure' OR CHF) | 2,612,296 | 336,727 |
| #2 | HIV/AIDS | TS=(AIDS OR ‘Acquired Immunodeficiency Syndrome*’ OR ‘Acquired Immune Deficiency Syndrome*’ OR ‘Acquired Immuno-Deficiency Syndrome*’ OR ‘Acquired Immuno Deficiency Syndrome*’ OR HIV OR ‘Human Immunodeficiency Virus*’ OR ‘Human Immuno Deficiency Virus*’ OR ‘Human Immuno-Deficiency Virus*’ OR ‘AIDS Virus*’ OR ‘Acquired Immune Deficiency Syndrome Virus*’ OR ‘Acquired Immunodeficiency Syndrome Virus*’ OR ‘HIV/AIDS’) | 987,551 | 153,556 |
| #3 | Ethiopia | TS=(Ethiop* OR Afar OR Amhara OR Benishangul* OR Gumuz* OR Gambel* OR Harar* OR Oromia* OR Somali* OR SNNP* OR 'Southern nation nationalit*' OR Tigra* Or 'Addis Ababa' OR 'Dire Dawa') | 65,742 | 19,971 |
| **#4** | **Final** | **#1 AND #2 AND #3** | **150** | **60** |

**Africa Index Medicus**

| Search | Key word (concepts) | Search string | Items found (13/11/2023) | Items found (13/03/2026) |
| --- | --- | --- | --- | --- |
| #1 | Cardiovascular disease | ((mh:C14.240* OR mh:C16.131.240*) OR (tw:("Cardiovascular disease*" OR "Cardio-vascular disease*" OR "Cardio vascular disease*" OR "Cardiovascular illness*" OR "Cardio-vascular illness*" OR "Cardio vascular illness*" OR "Cardiovascular mortalit*" OR "Cardio-vascular mortalit*" OR "Cardio vascular mortalit*" OR "Cardiac disease*" OR "Coronary disease*" OR "Cardiac arrest*" OR "Heart disease*" OR "Myocardial infarction" OR Stroke OR "Artery disease*" OR "Vascular disease*" OR Hypertensi* OR Aneurysm OR Arrhythmia OR Atherosclerosis OR Heart failure OR CVD OR "high blood pressure" OR CHF))) AND (db:("AIM")) | 1,671 | 65 |
| #2 | HIV/AIDS | ((mh:B04.820.650.589.650.350* OR mh:C01.221.250.875.040 OR mh:C01.221.812.640.400.040 OR mh:C01.778.640.400.040 OR mh:C01.925.782.815.616.400.040 OR mh:C01.925.813.400.040 OR mh:C01.925.839.040 OR mh:C12.100.937.640.400.040 OR mh:C20.673.480.040) OR (tw:(AIDS OR "Acquired Immunodeficiency Syndrome*" OR "Acquired Immune Deficiency Syndrome*" OR "Acquired Immuno-Deficiency Syndrome*" OR "Acquired Immuno Deficiency Syndrome*" OR HIV OR "Human Immunodeficiency Virus*" OR "Human Immuno Deficiency Virus*" OR "Human Immuno-Deficiency Virus*" OR "AIDS Virus*" OR "Acquired Immune Deficiency Syndrome Virus*" OR "Acquired Immunodeficiency Syndrome Virus*" OR "HIV/AIDS"))) AND (db:("AIM")) | 2,779 | 38 |
| #3 | Ethiopia | ((mh:Z01.058.290.120.310*) OR (tw:(Ethiop* OR Afar OR Amhara OR Benishangul* OR Gumuz* OR Gambel* OR Harar* OR Oromia* OR Somali* OR SNNP* OR "Southern nation nationalit*" OR Tigra* Or "Addis Ababa" OR "Dire Dawa"))) AND (db:("AIM")) | 566 | 25 |
| **#4** | **Final** | **#1 AND #2 AND #3** | **4** | **2** |

**ProQuest**

| Search | Key word (concepts) | Search string | Items found (13/11/2023) | Items found (13/03/2026) |
| --- | --- | --- | --- | --- |
| #1 | Cardiovascular disease | (noft("cardiovascular disease*") OR noft("Cardio-vascular disease*") OR noft("Cardio vascular disease*") OR noft("Cardiovascular illness*") OR noft("Cardio-vascular illness*") OR noft("Cardio vascular illness*") OR noft("cardiovascular mortality") OR noft("Cardio-vascular mortalit*") OR noft("Cardio vascular mortalit*") OR noft("cardiac disease") OR noft("cardiac diseases") OR noft("Coronary disease*") OR noft("cardiac arrest") OR noft("cardiac arrests") OR (noft("heart disease") OR noft("heart diseases")) OR noft("Myocardial infarction") OR noft(Stroke) OR noft("artery disease") OR noft("vascular disease") OR noft("vascular diseases") OR noft(Hypertensi*) OR noft(Aneurysm) OR noft(Arrhythmia) OR noft(Atherosclerosis) OR noft(Heart failure) OR noft(CVD) OR noft("high blood pressure") OR noft(CHF)) | 75,505 | 378,382 |
| #2 | HIV/AIDS | (noft(AIDS) OR noft("Acquired Immunodeficiency Syndrome*") OR noft("Acquired Immune Deficiency Syndrome*") OR noft("Acquired Immuno-Deficiency Syndrome*") OR noft("Acquired Immuno Deficiency Syndrome*") OR noft(HIV) OR noft("Human Immunodeficiency Virus*") OR noft("Human Immuno Deficiency Virus*") OR noft("Human Immuno-Deficiency Virus*") OR (noft("aids virus")) OR noft("Acquired Immune Deficiency Syndrome Virus*") OR noft("Acquired Immunodeficiency Syndrome Virus*") OR noft("HIV/AIDS")) | 114,343 | 431,025 |
| #3 | Ethiopia | (noft(Afar) OR noft(Amhara) OR noft(Benishangul*) OR noft(Gumuz*) OR noft(Gambel*) OR noft(Harar*) OR noft(Oromia*) OR noft(Somali*) OR noft(SNNP*) OR noft("Southern nation nationalit*") OR noft(Tigra*) Or noft("Addis Ababa") OR noft("Dire Dawa") OR noft(Ethiop*)) | 31,569 | 111,311 |
| **#4** | **Final** | **#1 AND #2 AND #3** | **2** | **91** |

**Addis Ababa University Institutional Repository**

| Search | Key word (concepts) | Search string | Items found (13/11/2023) | Items found (13/03/2026) |
| --- | --- | --- | --- | --- |
| #1 | Cardiovascular disease | ("Cardiovascular disease*" OR "Cardio-vascular disease*" OR "Cardio vascular disease*" OR "Cardiovascular illness*" OR "Cardio-vascular illness*" OR "Cardio vascular illness*" OR "Cardiovascular mortalit*" OR "Cardio-vascular mortalit*" OR "Cardio vascular mortalit*" OR "Cardiac disease*" OR "Coronary disease*" OR "Cardiac arrest*" OR "Heart disease*" OR "Myocardial infarction" OR stroke OR "Artery disease*" OR "Vascular disease*" OR Hypertensi* OR Aneurysm OR Arrhythmia OR Atherosclerosis OR Heart failure OR CVD OR "high blood pressure" OR CHF) | 1,442 | 172 |
| #2 | HIV/AIDS | (AIDS OR "Acquired Immunodeficiency Syndrome*" OR "Acquired Immune Deficiency Syndrome*" OR "Acquired Immuno-Deficiency Syndrome*" OR "Acquired Immuno Deficiency Syndrome*" OR HIV OR "Human Immunodeficiency Virus*" OR "Human Immuno Deficiency Virus*" OR "Human Immuno-Deficiency Virus*" OR "AIDS Virus*" OR "Acquired Immune Deficiency Syndrome Virus*" OR "Acquired Immunodeficiency Syndrome Virus*" OR "HIV/AIDS") | 1,594 | 114 |
| **#4** | **Final** | **#1 AND #2** | **83** | **7** |
