# Supplementary material for: Cardiovascular diseases among people living with HIV/AIDS in Ethiopia: A scoping review
Source: PLoS One. 2026 May 5;21(5):e0348283. doi: 10.1371/journal.pone.0348283 (PMC13143083; doi:10.1371/journal.pone.0348283)
Supplement: S3 File — (DOCX) [file pone.0348283.s003.docx]

**Supplementary file 3: List of excluded articles**

| **S.N.** | **Citation** | **Title** | **Exclusion reason** |
| --- | --- | --- | --- |
| 1 | Accorsi, s. et al, 2009 | Poverty, inequality and health: the challenge of the double burden of disease in a non-profit hospital in rural Ethiopia | Wrong patient population |
| 2 | Amare, H et al., 2021 | Predictors of glucose metabolism and blood pressure among Ethiopian individuals with HIV/AIDS after one-year of antiretroviral therapy | Not in CVD |
| 3 | Assefa, A. et al., 2023 | Prevalence and Factors Associated with Dyslipidemia Among People Living with HIV/AIDS on Follow-Up Care at a Tertiary Care Hospital in Ethiopia: A Cross-Sectional Study | Not in CVD |
| 4 | Belete, A. M. et al., 2021 | Serum Lipid Profiles of Patients Taking Efavirenz-Based Antiretroviral Regimen Compared to Ritonavir-Boosted Atazanavir with an Optimized Background at Zewditu Memorial Hospital, Addis Ababa, Ethiopia | Not in CVD |
| 5 | Duguma, N. et al., 2021 | Hematological parameters abnormalities and associated factors in HIV-positive adults before and after highly active antiretroviral treatment in Goba Referral Hospital, southeast Ethiopia: A cross-sectional study | Not in CVD |
| 6 | Gebrie, A., 2021 | The burden of metabolic syndrome in patients living with HIV/AIDS receiving care at referral hospitals of Northwest Ethiopia: A hospital-based cross-sectional study, 2019 | Not in CVD |
| 7 | Gleason, R. L et al., 2015 | Current Efavirenz (EFV) or Ritonavir-Boosted Lopinavir (LPV/r) Use Correlates with Elevate Markers of Atherosclerosis in HIV-Infected Subjects in Addis Ababa, Ethiopia | Not in CVD |
| 8 | Gleason, R. L et al., 2016 | Efavirenz and ritonavir-boosted lopinavir use exhibited elevated markers of atherosclerosis across age groups in people living with HIV in Ethiopia | Not in CVD |
| 9 | Huluka, D et al., 2019 | Prevalence and Risk Factors of Pulmonary Hypertension Among Adult HIV plus Patients Followed at a Large Referral Hospital in Addis Ababa, Ethiopia | Confrence abstract |
| 10 | Kemal, A. et al 2020 | Dyslipidemia and Associated Factors Among Adult Patients on Antiretroviral Therapy in Armed Force Comprehensive and Specialized Hospital, Addis Ababa, Ethiopia | Not in CVD |
| 11 | Korem, M et al., 2018 | High Prevalence of Hypertension in Ethiopian and Non-Ethiopian HIV-Infected Adults | Wrong setting |
| 12 | Köse, E et al., 2021 | Bibliometric Analysis of HIV and Exercise Literature based on Scientific Studies from 1990-2020 | Wrong outcomes |
| 13 | Majonga, E. D et al., 2020 | Carotid intima media thickness in older children and adolescents with HIV taking antiretroviral therapy | Wrong setting |
| 14 | Majonga, E. D et al., 2018 | High prevalence of echocardiographic abnormalities in older HIV-infected children taking antiretroviral therapy | Wrong setting |
| 15 | Mariam, A. G et al., 2012 | Clinical and neuroimaging profile of HIV-I encephalopathy in infancy and childhood in a Sub-saharan african country | Wrong setting |
| 16 | Melaku, T et al., 2019 | Immunologic restoration of people living with human immunodeficiency virus on highly active anti-retroviral therapy in Ethiopia: The focus of chronic non-communicable disease co-morbidities | Not in CVD |
| 17 | Memon, N et al., 2022 | Uremic pneumonitis: A forgotten etiology of DAH | Confrence abstract |
| 18 | Miller, R. F et al., 2013 | Cardiac Disease in Adolescents With Delayed Diagnosis of Vertically Acquired HIV Infection | Wrong setting |
| 19 | Misganaw, A et al., 2012 | The Double Mortality Burden Among Adults in Addis Ababa, Ethiopia, 2006-2009 | Wrong patient population |
| 20 | Moller, S. P et al., 2020 | HIV and metabolic syndrome in an Ethiopian population | Not in CVD |
| 21 | Rosebush, J. C et al., 2014 | Preclinical atherosclerosis in Eastern Africa: Results from a pediatric ethiopian cohort | Confrence abstract |
| 22 | Tadewos, A et al., 2012 | Prevalence of dyslipidemia among HIV-infected patients using first-line highly active antiretroviral therapy in Southern Ethiopia: a cross-sectional comparative group study | Not in CVD |
| 23 | Talargia et al., 2021 | Thrombocytopenia and associated factors among HIV infected patients in pre-and post-anti-retroviral therapy, North East Ethiopia | Not in CVD |
| 24 | Tegegne, K. D et al., 2023 | Prevalence and factors associated with hypertension among peoples living with HIV in East Africa, a systematic review and meta-analysis | Wrong setting |
| 25 | Tilahun, A et al 2022 | Prevalence and predictors of dyslipidemia among HAART treated and HAART naive HIV positive clients attending Debre Tabor Hospital, Debre Tabor, Ethiopia | Not in CVD |
| 26 | Ullerup-Aagaard et al., 2012 | Disseminated B-cell lymphoma with cardial involvement in an HIV positive patient | Confrence abstract |
| 27 | Woldu, M et al., 2022 | Biomarkers and Prevalence of Cardiometabolic Syndrome Among People Living With HIV/AIDS, Addis Ababa, Ethiopia: A Hospital-Based Study | Not in CVD |
| 28 | Woldu, M et al., 2023 | Cardiometabolic syndrome in HIV-positive and HIV-negative patients at Zewditu Memorial Hospital, Addis Ababa, Ethiopia: a comparative cohort study | Not in CVD |
| 29 | Badacho, A et al., 2023 | Sustainability of integrated hypertension and diabetes with HIV care for people living with HIV at primary health care in South Ethiopia: implication for integration | Wrong outcomes |
| 30 | Badacho, A et al., 2023 | Facilitators and barriers to integration of noncommunicable diseases with HIV care at primary health care in Ethiopia: a qualitative analysis using CFIR | Wrong outcomes |
| 31 | Badacho A et al., 2024 | Lived experiences of people living with HIV and hypertension or diabetes access to care in Ethiopia: a phenomenological study | Wrong outcomes |
