# Supplementary material for: Cardiovascular diseases among people living with HIV/AIDS in Ethiopia: A scoping review
Source: PLoS One. 2026 May 5;21(5):e0348283. doi: 10.1371/journal.pone.0348283 (PMC13143083; doi:10.1371/journal.pone.0348283)
Supplement: Attachment — Submitted filename: renamed_cfd11.pdf [file pone.0348283.s004.pdf]

Review for the article titled; "Relationship between Cardiovascular Diseases and HIV/AIDS in Ethiopia: A Scoping Review."

Thank you for allowing me to review such an interesting article. It is a well conducted scoping review. Please see my comments below:

1. The last search date was conducted in November 2023. It was more than six months ago. I suggest the authors update their search to current.
2. For readers not familiar with Ethiopia; kindly provide the population size so that the 1% HIV prevalence can provide more clarity on numbers affected. Compared to most of Sub Saharan Africa, this is a low prevalence.
3. Was the scoping review registered with any scoping review registry? If yes, please include the registration link on the manuscript. If not, please give a reason.
4. Line 31: a prior should be "a priori"
5. Line 42/42: "Better study design" - I suggest you reword to "more robust study designs"
6. Line 101/102: The authors say meta analysis was impossible due to differences regarding population, methods, and outcomes: please elaborate what were the exact differences ?
7. Line 113-114 : You can reference this statement. Could these be the regions that are mostly affected by HIV/AIDS?
8. Include the total sample size represented by the review.

9. Table 1: It would be more informative to include the sex distribution (e.g %females). Please include age range, indicate whether the study area is urban or rural setting
10. The authors found that over 60% of the studies were on hypertension. Because of this, it will be important to incorporate the definitions of hypertension in the included studies: the differences in measurement methods utilised and any other notable differences. Is there any data provided on whether study participants were on Blood pressure medication or not?
    Due to the importance of behavioural risk factors, I suggest authors include a summary of risk factors represented and include prevalence/incidence if included in the articles. For now, the review shows that studies have included these but it would be helpful to see the numbers.

    Line 128-129: Reference the minimum and maximum hypertension prevalences with respective studies

    Line 207 - 209: “Therefore, some institutions may be better than others in diagnosing hypertension, which might affect the prevalence of hypertension in that institution.” This needs to be explained. It is quite vague at the moment. What makes other institutions better than others? Are the referral hospitals better equipped or resourced than others?

    Line 248 : Not all studies were cross sectional. Please change this

Note: Is hypertension a disease or a medical condition?

Line 253-254: “The results of this study offer valuable information for decision-makers and all HIV-related healthcare providers, suggesting the inclusion of blood pressure assessment in primary prevention efforts.” - Does Ethiopia not routinely screen patients for hypertension when they visit the health facility? If this is the case you could mention it in the manuscript.

Limitations:

Include the limitation in restricting the language to English
