# Supplementary material for: Cardiovascular diseases among people living with HIV/AIDS in Ethiopia: A scoping review
Source: PLoS One. 2026 May 5;21(5):e0348283. doi: 10.1371/journal.pone.0348283 (PMC13143083; doi:10.1371/journal.pone.0348283)
Supplement: Attachment — Submitted filename: Errega et al 2025-2.pdf [file pone.0348283.s007.pdf]

Thank you to the authors for addressing my comments. Please find my comments below:

1. Line 153 - Recheck…. T should be The

2. Line 170 -174 is repeated from 176 - 182.

3. Line 275, remove the second ‘’the

4. Some of the databases indicated in your supplementary material section are not mentioned in the text, e.g ProQuest and your search strategies for Google scholar and Cochrane are not indicated. There are inconsistencies on the database searches;recheck the text, prisma diagram and search strategies.The Prisma diagram does not include some Google scholar search

5. I recommend that you register your scoping review on Open Science Framework, if possible
