# Supplementary material for: Cardiovascular diseases among people living with HIV/AIDS in Ethiopia: A scoping review
Source: PLoS One. 2026 May 5;21(5):e0348283. doi: 10.1371/journal.pone.0348283 (PMC13143083; doi:10.1371/journal.pone.0348283)
Supplement: Attachment — Submitted filename: PLOS one review.docx [file pone.0348283.s005.docx]

PLOS one review

Relationship between Cardiovascular Diseases and HIV/AIDS in Ethiopia: A Scoping Review

The title is too general. A study population is required in a title.

Abstract

- Methods: which framework was used to guide the review?
- What search strategy was used to search literature in electronic databases
- Which databases were selected and why?
- What was the duration of the search
- What was used to document the selection process

What are the strengths and limitations of this review?

Introduction/limitation

The introduction needs to be longer, and it needs to be clarified why there is a need for a scoping review. There is no data or statistics that support the need for the review.

Methodology

Clarity and structure of the methodology: the review is not well-organized, and it isn't easy to follow the steps. All the steps need to be more comprehensive.

The methodology needs to be longer, and important review steps must be included.

The authors must identify and describe the scoping review framework used to conduct this review.

What were the research questions for the scoping review?

Data source and search strategy: The article needs to be longer, and the authors have not used grey literature; the way I ask why?

*Eligibility criteria:*

*Which co-elements have the authors used to assess the eligibility of the research questions?*

Identifying relevant studies

The review needs to be more comprehensive. For instance, the authors identified relevant studies only from published literature databases. The grey literature was not considered,

Which keywords and terms were used to search for relevant studies from the electronic databases

Did the authors limit the search to dates? In other words, what were the years? Did they limit the years of the publications?

What did the authors use to facilitate the literature search, incorporating study topics, author information, and publication dates?

Which range of study designs were considered and included in the review

Discussion: the need major revision; the current discussion is the repetition of the results and

Conclusions and Recommendations

What is the conclusion of the study?

What are practical recommendations for future research or public health interventions?
