# Supplementary material for: Cardiovascular diseases among people living with HIV/AIDS in Ethiopia: A scoping review
Source: PLoS One. 2026 May 5;21(5):e0348283. doi: 10.1371/journal.pone.0348283 (PMC13143083; doi:10.1371/journal.pone.0348283)
Supplement: Attachment — Submitted filename: Response to Reviewers.docx [file pone.0348283.s009.docx]

Reviewers' comments:

Reviewer's Responses to Questions

**Comments to the Author**

1. Is the manuscript technically sound, and do the data support the conclusions?

The manuscript must describe a technically sound piece of scientific research with data that supports the conclusions. Experiments must have been conducted rigorously, with appropriate controls, replication, and sample sizes. The conclusions must be drawn appropriately based on the data presented.

Reviewer #1: Yes

Reviewer #2: Partly

2. Has the statistical analysis been performed appropriately and rigorously?

Reviewer #1: N/A

Reviewer #2: N/A

3. Have the authors made all data underlying the findings in their manuscript fully available?

The [PLOS Data policy](http://c05y1x9s.r.us-east-2.awstrack.me/L0/http:%2F%2Fwww.plosone.org%2Fstatic%2Fpolicies.action%23sharing/1/010f0192908ba8a5-3ff5f1cc-5b30-4cfc-be6a-9835b3969d32-000000/9bIp7jNSQD6SLsGOdIBuL1g2ImI=180) requires authors to make all data underlying the findings described in their manuscript fully available without restriction, with rare exception (please refer to the Data Availability Statement in the manuscript PDF file). The data should be provided as part of the manuscript or its supporting information, or deposited to a public repository. For example, in addition to summary statistics, the data points behind means, medians and variance measures should be available. If there are restrictions on publicly sharing data—e.g. participant privacy or use of data from a third party—those must be specified.

Reviewer #1: Yes

Reviewer #2: No

4. Is the manuscript presented in an intelligible fashion and written in standard English?

PLOS ONE does not copyedit accepted manuscripts, so the language in submitted articles must be clear, correct, and unambiguous. Any typographical or grammatical errors should be corrected at revision, so please note any specific errors here.

Reviewer #1: Yes

Reviewer #2: Yes

5. Review Comments to the Author

Please use the space provided to explain your answers to the questions above. You may also include additional comments for the author, including concerns about dual publication, research ethics, or publication ethics. (Please upload your review as an attachment if it exceeds 20,000 characters)

Reviewer #1: Authors just need to read through through the article to check the very few typos. I highlighted what I could find n the report. My main concern is that the last search was conducted over six months ago and needs to be updated. Also further detail on differences in population and definitions of hypertension need to be provided. Some mention of current practice at health care facilities in terms of what they routinely screen for will be informative.

Reviewer #2: The manuscript lacks a conclusion, and there is no need for statistical analysis. Additionally, the authors have not made the data fully available. However, it is worth noting that the manuscript is written in standard English.The authors must identify and describe the scoping review framework used to conduct this review.
What were the research questions for the scoping review?Clarity and structure of the methodology: the review is not well-organized, and it isn't easy to follow the steps. All the steps need to be more comprehensive.

6. PLOS authors have the option to publish the peer review history of their article ([what does this mean?](https://c05y1x9s.r.us-east-2.awstrack.me/L0/https:%2F%2Fjournals.plos.org%2Fplosone%2Fs%2Feditorial-and-peer-review-process%23loc-peer-review-history/1/010f0192908ba8a5-3ff5f1cc-5b30-4cfc-be6a-9835b3969d32-000000/1U8U1ZB4sbJJxFXJfXFxxRLWlIQ=180)). If published, this will include your full peer review and any attached files.


If you choose “no”, your identity will remain anonymous but your review may still be made public.


**Do you want your identity to be public for this peer review?** For information about this choice, including consent withdrawal, please see our [Privacy Policy](https://c05y1x9s.r.us-east-2.awstrack.me/L0/https:%2F%2Fwww.plos.org%2Fprivacy-policy/1/010f0192908ba8a5-3ff5f1cc-5b30-4cfc-be6a-9835b3969d32-000000/6jm_VYqvzJ9ysG9ZojGIWUNvQI4=180).

Reviewer #1: No

Reviewer #2: No

**Reviewers' Comments to Author:**

**Reviewer: 1**

Thank you for allowing me to review such an interesting article. It is a well

conducted scoping review.

Thank you for your constructive feedback.

Please see my comments below:

1. The last search date was conducted in November 2023. It was more than six months ago. I suggest the authors update their search to current.

We appreciate the reviewer’s valuable observation regarding the timeliness of our literature search. We agree that an updated search is essential to ensure the inclusion of the most recent and relevant studies. Accordingly, we commit to conducting an updated search prior to the final publication of the manuscript to capture any newly published literature and maintain the review’s currency and comprehensiveness.

2. For readers not familiar with Ethiopia; kindly provide the population size so that the 1% HIV prevalence can provide more clarity on numbers affected. Compared to most of Sub Saharan Africa, this is a low prevalence.

Thank you for your comment. It has been corrected as follows as per the recommendation: *“Around 1% of the population (around 1.4 million people) is still affected…”*

3. Was the scoping review registered with any scoping review registry? If yes, please include the registration link on the manuscript. If not, please give a reason.

4. Line 31: a prior should be “a priori”

Thank you for your feedback. It has been corrected as per the recommendation.

5. Line 42/42: “Better study design” - I suggest you reword to “more robust study designs”

Thank you for your feedback. It has been corrected as per the recommendation.

6. Line 101/102: The authors say meta-analysis was impossible due to differences regarding population, methods, and outcomes: please elaborate what were the exact differences?

Thank you for your comment. It has been corrected as follows as per the recommendation:

*“A narrative synthesis approach was used to summarize the findings due to substantial heterogeneity in study designs (e.g., cohort, cross-sectional, case report), populations (e.g., HAART versus non-HAART patients), outcomes (e.g., hypertension, ischemic stroke, ECG abnormalities), and analytical methods. As such, meta-analysis was not feasible.”*

7. Line 113-114: You can reference this statement. Could these be the regions that are mostly affected by HIV/AIDS?

Thank you for your feedback. It has been corrected as per the recommendation.

8. Include the total sample size represented by the review.

Thank you for your comment. It has been corrected as follows as per the recommendation: *“The total sample size represented in this review comprises 6,633 participants….”*

9. Table 1: It would be more informative to include the sex distribution (e.g. %females). Please include age range, indicate whether the study area is urban or rural setting

Thank you for this constructive feedback. In response, we have extracted and included age, gender, and residential characteristics of participants.

*“Although not all studies reported the age, gender, and residential characteristics of their participants, available data indicate that approximately 62% were female and 74.5% resided in urban areas. Nearly all studies focused on adult populations, with the exception of two studies: one was a case report involving an infant (20), and the other exclusively included fetuses (21).”*

10. The authors found that over 60% of the studies were on hypertension. Because of this, it will be important to incorporate the definitions of hypertension in the included studies: the differences in measurement methods utilised and any other notable differences. Is there any data provided on whether study participants were on Blood pressure medication or not?

Thank you for this constructive feedback. In response, we have extracted and included the definition of hypertension across the included studies.

*“The definition of hypertension varied across the included studies. Five studies defined hypertension as a blood pressure of ≥140/90 mmHg, while six studies used the same threshold or included individuals taking antihypertensive medication. The remaining three studies applied a lower threshold, defining hypertension as a blood pressure of ≥130/80 mmHg.”*

Due to the importance of behavioural risk factors, I suggest authors include a summary of risk factors represented and include prevalence/incidence if included in the articles. For now, the review shows that studies have included these, but it would be helpful to see the numbers.

We appreciate the reviewer’s insightful comment regarding the importance of behavioral risk factors for cardiovascular disease among people living with HIV/AIDS. In our initial inclusion criteria, we focused on studies that reported on the prevalence, incidence, or clinical outcomes of cardiovascular diseases and excluded those that assessed only risk factors without reporting cardiovascular disease outcomes. This was done to ensure the review remained centered on cardiovascular disease burden among PLWHA.

Line 128-129: Reference the minimum and maximum hypertension prevalences with respective studies

Thank you for your feedback. It has been corrected as per the recommendation. We have now cited the minimum and maximum hypertension prevalences with respective studies.

Line 207 - 209: “Therefore, some institutions may be better than others in diagnosing hypertension, which might affect the prevalence of hypertension in that institution.” This needs to be explained. It is quite vague at the moment. What makes other institutions better than others? Are the referral hospitals better equipped or resourced than others?

Thank you for your feedback. We agree that the sentence was vague. Following also feedback from the other reviewer, we have not rewritten the discussion section. We believe the new discussion presents a more coherent and is easier to read.

Line 248: Not all studies were cross sectional. Please change this

Thank you for your feedback. We have now changed this section with *“Most of the included primary studies were cross-sectional ....”*

Note: Is hypertension a disease or a medical condition?

Thank you for your question. Hypertension is primarily considered a medical condition, but it is also widely recognized as a chronic or non-communicable disease (NCD), especially in clinical and public health contexts. While the term "medical condition" broadly refers to any state that requires medical attention, "disease" denotes a more specific pathological process. Because hypertension can lead to serious complications such as heart disease, stroke, and kidney failure, it is often categorized as a chronic disease in the medical literature. Public health organizations like the World Health Organization and the CDC commonly refer to hypertension as an NCD due to its long-term impact on health and its association with other cardiovascular diseases. Therefore, both terms are accurate, but we believe referring to hypertension as a chronic or non-communicable disease is more precise in academic and health-related discussions.

Line 253-254: “The results of this study offer valuable information for decision-makers and all HIV-related healthcare providers, suggesting the inclusion of blood pressure assessment in primary prevention efforts.” - Does Ethiopia not routinely screen patients for hypertension when they visit the health facility? If this is the case you could mention it in the manuscript.

Limitations:

Include the limitation in restricting the language to English

Thank you for your feedback. It has been corrected as per the recommendation. We have now added the following sentence in the limitation section.

*“Additionally, the review was restricted to studies published in English, which raises the possibility that relevant studies published in other languages may have been inadvertently not included.”*

**Reviewer: 2**

The title is too general. A study population is required in a title.

Thank you for your comment regarding the title. We agree that the original title was too general. To address this, we have revised the title to better reflect the study population and the specific focus of our review. The new title, *"Cardiovascular Diseases among People Living with HIV/AIDS in Ethiopia: A Scoping Review,"* more clearly identifies the target population and the scope of the study. This revision aims to improve the clarity and specificity of the title, ensuring it accurately conveys the focus of our research. Thank you for your valuable feedback.

Abstract

- Methods: which framework was used to guide the review?
- What search strategy was used to search literature in electronic databases
- Which databases were selected and why?
- What was the duration of the search
- What was used to document the selection process

What are the strengths and limitations of this review?

We appreciate the reviewer’s comments on the abstract and have revised it accordingly to enhance clarity and completeness. In response to the feedback, we have explicitly stated the methodological framework used (Arksey and O’Malley, enhanced by Levac et al.), clarified the databases searched (PubMed, Scopus, Google Scholar, Web of Science, African Journals Online, and Cochrane Library), and justified their inclusion based on relevance and accessibility. We also specified the search timeline (up to November 2023) and added that a PRISMA flow diagram was used to document the selection process.

Additionally, we now include the strengths and limitations of the review, particularly the comprehensiveness of the search and the limitations of cross-sectional and institution-based data. We have restructured the abstract into a cohesive paragraph form and ensured it remains within the 300-word limit. We believe these revisions address all the reviewer’s concerns and have strengthened the abstract’s clarity and informativeness.

Introduction/limitation

The introduction needs to be longer, and it needs to be clarified why there is a need for a scoping review. There is no data or statistics that support the need for the review.

Thank you for this constructive feedback. We agree that the original introduction was too brief and did not provide sufficient justification for the need for a scoping review. In response, we have substantially revised and expanded the introduction section. The revised version now includes a clearer rationale for conducting a scoping review, emphasizing the fragmented and varied nature of the existing literature on HIV and CVDs in the Ethiopian context. We believe the new introduction presents a more coherent and compelling case for the relevance and timeliness of this review.

Methodology

Clarity and structure of the methodology: the review is not well-organized, and it isn't easy to follow the steps. All the steps need to be more comprehensive.

We appreciate the reviewer’s observation. We have now reorganized the methodology section into a more structured format that aligns with recognized scoping review frameworks. The revised section is organized into key steps, including search strategy, eligibility criteria, study selection, data charting, and data synthesis, and each step has been described in more detail to enhance clarity and comprehensiveness.

The methodology needs to be longer, and important review steps must be included.

Thank you for this constructive feedback. In response, we have expanded the methodology section to include detailed descriptions of each step in the scoping review process.

The authors must identify and describe the scoping review framework used to conduct this review.

Thank you for this helpful suggestion. We have now explicitly stated that our review followed the Arksey and O’Malley (2005) framework, further enhanced by Levac et al. (2010) and the Joanna Briggs Institute (JBI) guidance. These sources are now cited in the methodology section to clarify the theoretical underpinnings of our review process.

What were the research questions for the scoping review?

Thank you for your question. The review was guided by the following research questions: (1) What are the types of studies conducted on PLWHA concerning cardiovascular diseases in Ethiopia? (2) What is the prevalence/incidence of cardiovascular diseases in PLWHA in Ethiopia? And (3) What are the key risk factors associated with cardiovascular diseases among this population? Please note that this is included at the end of the introduction section.

Data source and search strategy: The article needs to be longer, and the authors have not used grey literature; the way I ask why?

Thank you for pointing this out. We have expanded this section and clarified that grey literature was indeed considered. We searched the Addis Ababa University digital thesis repository and conducted a manual search of bibliographies of relevant studies to identify grey literature. This is now explicitly stated in the revised methodology.

*Eligibility criteria:*

*Which co-elements have the authors used to assess the eligibility of the research questions?*

We appreciate the reviewer’s comment. We have clarified in the revised section that the inclusion criteria were aligned with the Population–Concept–Context (PCC) framework recommended for scoping reviews by the Joanna Briggs Institute. Specifically, our population was PLWHA, the concept was cardiovascular disease, and the context was Ethiopia. This has been added to enhance transparency in study selection.

Identifying relevant studies

The review needs to be more comprehensive. For instance, the authors identified relevant studies only from published literature databases. The grey literature was not considered,

As noted above, this issue has been addressed. In addition to the five major databases, **grey literature** sources such as university repositories and reference lists were screened manually. We have made this explicit in the revised methodology under the section describing sources searched.

Which keywords and terms were used to search for relevant studies from the electronic databases

Thank you for your question. The core search terms and phrases were 'cardiovascular disease,' 'HIV,' AIDS,' and 'Ethiopia'

Did the authors limit the search to dates? In other words, what were the years? Did they limit the years of the publications?

We have clarified that no date restrictions were applied during the search. The search covered literature from inception to November 13, 2023. This is now clearly stated in the methodology.

What did the authors use to facilitate the literature search, incorporating study topics, author information, and publication dates?

We have clarified that a structured search strategy using both MeSH terms and free-text keywords was developed for PubMed and adapted for other databases. The strategy incorporated key terms related to cardiovascular disease, HIV/AIDS, and Ethiopia. We also indicated that EndNote was used for reference management, and Covidence was used for screening and managing the review workflow. Details of the search terms are provided in Supplementary File 1.

Which range of study designs were considered and included in the review

We thank the reviewer for this important point. We have clarified that all study designs were eligible for inclusion, including cross-sectional studies, cohort studies, case-control studies, and case reports. Only commentaries, editorials, protocols, and other non-primary research formats were excluded. This clarification has been added to the eligibility criteria section.

Discussion: the need major revision; the current discussion is the repetition of the results and

Thank you for this constructive feedback. We have thoroughly revised the Discussion section to address this concern. In the revised version, we moved beyond a simple restatement of results and instead provided an in-depth interpretation of the findings within the broader context of the literature. We synthesized insights from both regional and global studies to compare and contrast the prevalence and risk factors for cardiovascular diseases among PLWHA in Ethiopia. Additionally, we discussed the clinical, policy, and research implications of our findings, including recommendations for integrating cardiovascular screening into routine HIV care, the need for community-based studies, and the limitations of the current evidence base. The revised discussion now offers a more critical and analytical narrative aligned with the purpose of a scoping review and avoids redundancy with the results section.

We hope that the revised discussion meets the expected academic standard and addresses your concerns. Thank you once again for helping improve the quality of our manuscript.

Conclusions and Recommendations

What is the conclusion of the study?

Thank you for highlighting the need to strengthen the conclusion section. We have now revised the manuscript to include a clear and concise conclusion that encapsulates the key findings and their implications. The revised conclusion summarizes the prevalence and associated factors of cardiovascular diseases among PLWHA in Ethiopia, emphasizes the need for integrated care models, and highlights the importance of addressing research gaps through community-based and longitudinal studies. Furthermore, we have included actionable recommendations for healthcare providers, policymakers, and researchers to enhance cardiovascular care and prevention for PLWHA in Ethiopia.

What are practical recommendations for future research or public health interventions?

Thank you for your comment. In response, we have revised the discussion and conclusion sections to include practical recommendations for future research and public health interventions.
