# Supplementary material for: Cardiovascular diseases among people living with HIV/AIDS in Ethiopia: A scoping review
Source: PLoS One. 2026 May 5;21(5):e0348283. doi: 10.1371/journal.pone.0348283 (PMC13143083; doi:10.1371/journal.pone.0348283)
Supplement: Attachment — Submitted filename: PLOSEONE REVIEW.docx [file pone.0348283.s008.docx]

PLOSEONE REVIEW

Date: 06/08/2025

TITLE: CARDIOVASCULAR DISEASES AMONG PEOPLE LIVING WITH HIV

The review requires language editing. It's evident that authors used AI for editing; however, there are still grammatical errors in sentence construction. For instance, scientific writing requires authors to avoid starting sentences with a preposition, and another example, starting a sentence with “ Global” the correct way is “ The global”; this error can be corrected by Grammarly software

The first part of the scoping is well written, and the authors have responded well to the previous queries; however, there is a need for major revision for the methodology and discussion of the review.

**Methodology**

The authors claim they used the methodological framework proposed by Arksey and O’Malley (14), which was later refined by Levac et al. (15) and aligned with the Joanna Briggs Institute (JBI) guidelines for scoping reviews; however, the subheadings that are recommended by the framework are not visible.

The methodological framework has the following stages, which are required to fulfil the requirements of the scoping review:

1. Stage one: identification of the research question/s: the authors haven’t written their research question/s, and it's unclear what the purpose of the review is. Within this stage, it is a requirement to describe the eligibility of the research question/s; also, the authors have not indicated the framework that they used for the scoping review, eligibility criteria belong to stage one of the frameworks

**The authors must correct the order of the following stages**

2. Stage two: identifying relevant studies
3. Study selection and appraisal
4. Collating, summarizing and reporting of the results

***Results***

*This is the first time I see Factors associated with cardiovascular disease among PLWHA, was it one of the aims of conducting the review? The title of the study doesn’t mention the factors. Please explain why you decided to include the factors*

Discussion

The discussion must be structured according to the research question/s and the results of the review.

What are the strengths and limitations of this review?
