# Supplementary material for: Cardiovascular diseases among people living with HIV/AIDS in Ethiopia: A scoping review
Source: PLoS One. 2026 May 5;21(5):e0348283. doi: 10.1371/journal.pone.0348283 (PMC13143083; doi:10.1371/journal.pone.0348283)
Supplement: Attachment — Submitted filename: Response_to_reviewers_auresp_2.docx [file pone.0348283.s010.docx]

**Reviewer 1**

Thank you to the authors for addressing my comments.

Thank you very much for the encouraging feedback.

Please find my comments below:

1. Line 153 - Recheck…. T should be The

2. Line 170 -174 is repeated from 176 - 182.

3. Line 275, remove the second ‘’the

Thank you for your feedback and apologies for the typos. The manuscript has now been thoroughly copy edited throughout with a grammar software. Also, the repeated sentences were deleted.

4. Some of the databases indicated in your supplementary material section are not mentioned in the text, e.g ProQuest and your search strategies for Google scholar and Cochrane are not indicated. There are inconsistencies on the database searches; recheck the text, Prisma diagram and search strategies. The Prisma diagram does not include some Google scholar search.

Thank you for your helpful suggestion. Search was conducted on the following databases: PubMed, EMBASE, ProQuest, Global Index Medicus, and Web of Science. There was a mistake on the abstract section on the list of databases we searched. Apologies for the mistake we have made and we have now corrected the mistake. The text, Prisma diagram and search strategies are now synchronized.

5. I recommend that you register your scoping review on Open Science Framework, if possible

Thank you for your recommendation. We are working on getting the study registered on OSF.

**Reviewer 2**

The review requires language editing. It's evident that authors used AI for editing; however, there are still grammatical errors in sentence construction. For instance, scientific writing requires authors to avoid starting sentences with a preposition, and another example, starting a sentence with “ Global” the correct way is “ The global”; this error can be corrected by Grammarly software

Thank you for your feedback. The manuscript has now been thoroughly copy edited throughout with a grammar software.

The first part of the scoping is well written, and the authors have responded well to the previous queries; however, there is a need for major revision for the methodology and discussion of the review.

Thank you very much for the encouraging feedback and suggestions. Our responses to each point are provided below in a point-by-point format.

Methodology: The authors claim they used the methodological framework proposed by Arksey and O’Malley (14), which was later refined by Levac et al. (15) and aligned with the Joanna Briggs Institute (JBI) guidelines for scoping reviews; however, the subheadings that are recommended by the framework are not visible.

The methodological framework has the following stages, which are required to fulfil the requirements of the scoping review:

1. Stage one: identification of the research question/s: the authors haven’t written their research question/s, and it's unclear what the purpose of the review is. Within this stage, it is a requirement to describe the eligibility of the research question/s; also, the authors have not indicated the framework that they used for the scoping review, eligibility criteria belong to stage one of the frameworks

The authors must correct the order of the following stages

2. Stage two: identifying relevant studies
3. Study selection and appraisal
4. Collating, summarizing and reporting of the results

Thank you for your comment. The subheadings and the structure of the method section of the study has now been corrected based on the recommendation of the proposed framework: 1) Identifying the Research Question, 2) Identifying Relevant Studies, 3) Study selection and appraisal, 4) Data Charting process and 5) Collating, Summarizing, and Reporting the Results.

Results: This is the first time I see Factors associated with cardiovascular disease among PLWHA, was it one of the aims of conducting the review? The title of the study doesn’t mention the factors. Please explain why you decided to include the factors

Thank you for your question. Yes, identify factors associated with cardiovascular comorbidities in Ethiopian PLWHA was one of the objectives of the study and it is mentioned at the last paragraph of the introduction section. As a result one of the three research question was: What are the key factors associated with cardiovascular diseases among this population? The title of the study is modified following the feedback from reviewer on the first round of the review process.

Discussion: The discussion must be structured according to the research question/s and the results of the review.

Thank you for your feedback. The study was guided by three main objectives: (1) to describe the type and content of existing studies on cardiovascular diseases among people living with HIV/AIDS in Ethiopia; (2) to report the magnitude and distribution of cardiovascular diseases within this population; and (3) to identify factors associated with cardiovascular comorbidities among Ethiopian PLWHA.

Accordingly, we have structured the Discussion section around these three objectives. The first paragraph discusses the types of studies identified on cardiovascular diseases among people living with HIV/AIDS. The second paragraph presents the magnitude and distribution of cardiovascular diseases in this population, with particular emphasis on hypertension, which was the primary focus of most included studies. The third paragraph addresses the factors associated with cardiovascular diseases, again highlighting hypertension as the most frequently examined condition. We then discuss the implications of our findings for future research, clinical practice, and policy. Finally, the Discussion concludes with a paragraph summarizing the strengths and limitations of this review.

What are the strengths and limitations of this review?

Thank you for your question. Here are the strengths of the study. The study is the first in Ethiopia to summarize evidence on cardiovascular diseases in PLWHA. We tried to look at the relationship between HIV/AIDS and any cardiovascular diseases without restriction. We extensively searched both gray and peer-reviewed literature from 6 different sources. Additionally, there was no study design or time restriction in including articles.

Similarly, here are the limitation of the study. Most of the included primary studies were cross-sectional and institution-based studies. Therefore, the prevalence reported in those studies might not represent the community's prevalence. Additionally, the review was restricted to studies published in English, which raises the possibility that relevant studies published in other languages may have been inadvertently excluded. We recommend that future studies include participants from the community and with different study designs.

We have included the strength and limitation of the study at the last paragraph of the discussion.
