# Supplementary material for: Cardiovascular diseases among people living with HIV/AIDS in Ethiopia: A scoping review
Source: PLoS One. 2026 May 5;21(5):e0348283. doi: 10.1371/journal.pone.0348283 (PMC13143083; doi:10.1371/journal.pone.0348283)
Supplement: Attachment — Submitted filename: Response_to_reviewers_auresp_3.docx [file pone.0348283.s011.docx]

# **Cover Letter**

Date: 20-03-26

**Dr. Adetayo Olorunlana
Academic Editor**

**PLOS One**

**Re: Cardiovascular Diseases among People Living with HIV/AIDS in Ethiopia: A Scoping Review, PONE-D-24-23021R2**

**Dear Dr. Adetayo Olorunlana,**

We are grateful for the thorough review of our manuscript and for the insightful comments. We have now made changes to the manuscript based on the reviewers' comments. We also presented our point-by-point responses to the reviewers' comments.

Thank you again for considering our manuscript for publication in your journal.

Kind regards,

Yohannes Gebreegziabhere, on behalf of the authors

**Reviewers' comments:**

**Reviewer #1:**

Line 33 – Please specify the date

*Thank you for your feedback. We have now revised it as suggested. See line 31 of the revised manuscript.*

Line 44 – Robust study designs. you can be more specific with this. Longitudinal studies would be more informative

*Thank you for your feedback. We have now revised it as suggested. See line 44 of the revised manuscript.*

Line 108 – We are already at the end of 2025. You can update your search, so that the results are as current as possible.

*Thank you for your feedback. We have now updated the search on March 13, 2026, and included seven additional papers. Considering this, changes were made across the manuscript.*

Line 131 – was the checking done by the same pr another reviewer?

*Thank you for your feedback. We have now explicitly indicated that checking for accuracy and completeness was done by another reviewer. See line 130 of the revised manuscript.*

Line 190/191 should be written for clarity

*Thank you for your feedback. The sentence is now rewritten for clarity as suggested.*

*“Most studies reported similar factors associated with hypertension. The most frequently reported factors were sociodemographic factors …”, Line 190*

Line 196 – it’s the second time that HAART is being written in full

*Thank you for your feedback. We have now revised it as suggested. See line 196 of the revised manuscript.*

Line 122: Woldu 2022, include “of” after prevalence for the first lines

*Thank you for your feedback. We have now copyedited the table, including fixing typos. See Table 2 for this change.*

Discussion – you can state what the definition of hypertension is followed for Ethiopia, and any differences in the measurement across studies. Was it resting blood pressure?

*We would like to thank the reviewer for this comment. Considering it, we have now included the working definition for hypertension in the discussion section. Thank you for the comment.*

*“Furthermore, according to the hypertension treatment guideline, in Ethiopia, hypertension is diagnosed if, on two visits on different days, systolic blood pressure is ≥140 mmHg and/or diastolic blood pressure is ≥90 mmHg on both days. However, the studies included in these reviews used various definitions of hypertension, which requires careful interpretation of the findings. “Line 251-255.*

Line 292: In this case the exclusion of articles by language is not inadvertent

Thank you for your feedback. We have now revised it as suggested. *See line 292 of the revised manuscript.*

Line 293’ specify on other study designs

Thank you for your feedback. We have now revised it as suggested. *See line 295 of the revised manuscript.*

Line 298 – recheck the spelling of behaviour

Thank you for your feedback. We have now copyedited the manuscript, including fixing typos and ensuring consistency. This also includes ensuring words like 'behaviour' are written in correct British English. We believe the manuscript is now easier to read.

You can conduct a critical appraisal to improve the paper

We acknowledge the reviewer's feedback and agree that a critical appraisal can improve the study's quality. However, as this is a scoping review and we included studies with different study designs, we could not conduct a critical appraisal.
